# Notes and News

**Published:** 1899-04-22

**Authors:** 


### Notes and News.

(Collected and Communicated.)

### HOSPITAL MEETINGS.

**Royal Eye Hospital, Southwark.—Festival Dinner, Friday, April 21st, at Whitehall Rooms, Hotel Metropole.**

**Metropolitan Hospital, Kingsland Road.—Festival Dinner, Monday, April 24th, Whitehall Rooms, Hotel Metropole.**

**Royal Hospital for Incurables, Putney Heath.—Festival Dinner, Tuesday, April 25th, Whitehall Rooms, Hotel Metropole.**

**London Lock Hospital and Rescue Home (late Asylum), Harrow Road, W.—Annual Meeting, at five p.m., on Thursday, April 27th, at Male Hospital, 91 Dean Street, Soho.**

The King of the Belgians, as Sovereign of the Congo Free State, has sent a handsome contribution towards the establishment of the London School of Tropical Medicine, which will entail the enlargement of the branch hospital of the Seamen's Hospital.

It is a pity that a more satisfactory account is not forthcoming of the funds contributed to Maidstone during the terrible typhoid epidemic. This is probably due to the fact that owing to the scare there was but little organisation at the outset, and that strict business methods of procedure were overlooked.

An interesting exhibition is now taking place on behalf of the Prince of Wales's Fund. It consists of a collection of forgeries, strange pictures, old coins, nicknacks, and lastly of a valuable Egyptian mummy. The collection belongs to Mr. Palmer, and is to be seen at 7, Catherine Street, Strand, for an entrance fee of one shilling.

The scheme for the establishment of a National Physical Laboratory is rapidly getting into shape. It is intended to enlarge and adapt the Kew observatory in the Old Deer Park at Richmond, besides erecting new buildings close at hand. For this purpose the Government will be asked for a grant of £12,000, and to provide a sum of £4,000 yearly for five years.

We are requested by the hon. secretary of the Jenner Society (Dr. Bond, Gloucester) to ask any of our readers who may be practising in localities in which the agitation against vaccination may be acute, either in form of newspaper correspondence or of impending lectures or debates, to be good enough to communicate with him. If newspapers are sent with reports the article to which it is desired to call attention should be marked.

Lord Lister has consented to become the first president of the St. Mary's Children's Hospital, at Plaistow, E.

The Johannesburg Hospital will become the recipient of £100 from the *Standard and Diggers' News* if it can be demonstrated that 25 per cent. of the signatures of the Uitlanders' petition to the Queen are genuine.

Mr. Frederic H. Madden, for 10 years secretary of the Medical School at St. Mary's Hospital, Paddington, has been appointed secretary to the Asylum for the Education of the Deaf and Dumb Children of the Poor, Old Kent Road, Surrey, and Margate, Kent, in succession to Mr. W. Resbury Few, resigned.

The new Lunacy Bill which has been introduced into the House of Lords by the Lord Chancellor provides for the shortening of the duration of the urgency order to four days. It also makes provision for the temporary care of patients in whom lunacy is not confirmed for a period not exceeding six months.

Recent reports on the subject of rabies in England are most hopeful. Some two years ago the muzzling order appeared to have effected little improvement, but the stringent manner in which the regulations have been enforced since then has undoubtedly been most effective. The last return for 14 weeks contains a clean record. In 1896, for the same period, no less than 2,107 cases were reported. Last year the number returned was eight.

Few will be found to grumble at the order for toothbrushes which the Watford Guardians have given, in order that the teeth of the children under their care may receive proper attention. Verily, it will be fortunate for those of the present generation who, instead of being left to the tender mercies of well-to-do but ignorant or careless parents, are cared for and educated out of the rates. The School Board carries out a system of intelligent teaching which would put most of the schools of the wealthy to shame, and now the hygienic regulations tend to become equally exemplary.

We regret to have to record the death of Sir William Roberts, M.D., F.R.S., F.R.C.P. He was born in 1830, and graduated B.A. at London University in 1851, becoming a member of the Royal College of Surgeons the same year, and taking the degree of M.D. at the London University in 1854. After being house surgeon at the Manchester Royal Infirmary for a short time he was appointed on the honorary staff, becoming physician to the infirmary at the age of 25. This appointment he held for nearly thirty years. He was the first professor of medicine in the Victoria University, and shortly after this, namely in 1885, he received the honour of knighthood. In 1866 he delivered the Gulstonian Lectures before the Royal College of Physicians, and in 1880 the Lumleian Lectures; in 1892 he was appointed Croonian Lecturer, and in 1897 he gave the Harveian oration before the college. After a long and successful career as a consulting physician in Manchester Sir William Roberts removed to London in 1889. In 1893 he proceeded to India as a member of the Royal Commission on Opium, and had recently been selected to serve on the statutory commission appointed to establish the University of London on its new footing.

